# Methods for enhancing the reproducibility of biomedical research findings using electronic health records

**DOI:** 10.1186/s13040-017-0151-7

**Published:** 2017-09-11

**Authors:** Spiros Denaxas, Kenan Direk, Arturo Gonzalez-Izquierdo, Maria Pikoula, Aylin Cakiroglu, Jason Moore, Harry Hemingway, Liam Smeeth

**Affiliations:** 1 Institute of Health Informatics, University College London, 222 Euston Road, London, NW1 2DA UK; 2 Farr Institute of Health Informatics Research, 222 Euston Road, London, UK; 3 The Francis Crick Institute, 1 Midland Road, London, NW1 1AT UK; 4 Institute of Biomedical Informatics, University of Pennsylvania, Richards Medical Research Laboratories, 3700 Hamilton Walk, Philadelphia, 19104 USA; 5 EHR Research Group, Department of Non-communicable Disease Epidemiology, London School of Hygiene and Tropical Medicine, Keppel Streeet, London, WC1E 7HT UK

**Keywords:** Electronic health records, Reproducibility, Transparency, Biomedical research

## Abstract

**Background:**

The ability of external investigators to reproduce published scientific findings is critical for the evaluation and validation of biomedical research by the wider community. However, a substantial proportion of health research using electronic health records (EHR), data collected and generated during clinical care, is potentially not reproducible mainly due to the fact that the implementation details of most data preprocessing, cleaning, phenotyping and analysis approaches are not systematically made available or shared. With the complexity, volume and variety of electronic health record data sources made available for research steadily increasing, it is critical to ensure that scientific findings from EHR data are reproducible and replicable by researchers. Reporting guidelines, such as RECORD and STROBE, have set a solid foundation by recommending a series of items for researchers to include in their research outputs. Researchers however often lack the technical tools and methodological approaches to actuate such recommendations in an efficient and sustainable manner.

**Results:**

In this paper, we review and propose a series of methods and tools utilized in adjunct scientific disciplines that can be used to enhance the reproducibility of research using electronic health records and enable researchers to report analytical approaches in a transparent manner. Specifically, we discuss the adoption of scientific software engineering principles and best-practices such as test-driven development, source code revision control systems, literate programming and the standardization and re-use of common data management and analytical approaches.

**Conclusion:**

The adoption of such approaches will enable scientists to systematically document and share EHR analytical workflows and increase the reproducibility of biomedical research using such complex data sources.

## Background

Electronic health records (EHR), data generated and captured during routine clinical care encounters across health care settings, have been recognized as an invaluable research resource [1, 2]. Increasingly, EHR are linked with genotypic data to enable precision medicine by examining how genetic variants influence susceptibility towards disease, validate drug targets, or modify drug response at a scale previous unobtainable. EHR research resources such as CALIBER [3], the Precision Medicine Cohort Initiative [4], the UK Biobank [5] and the eMERGE Network [6] enable researchers to investigate disease aetiology and prognosis [7–14] at unprecedented phenotypic depth and breadth by recreating the longitudinal patient pathway spanning genome to phenome and birth to death.

The replication of scientific findings using independent investigators, methods and data is the cornerstone of how published scientific claims are evaluated and validated by the wider scientific community [15–17]. Academic publications arguably have three main goals: a) to disseminate scientific findings, b) to persuade the community that the findings are robust and were achieved through rigorous scientific approaches and c) to provide a detailed description of the experimental approaches utilized. Peer-reviewed manuscripts should, in theory, describe the methods used by researchers in a sufficient level of detail as to enable other researchers to replicate the study.

A recent literature review [18] of research studies using national structured and linked UK EHR illustrated how a substantial proportion of studies potentially suffer from poor reproducibility: only 5.1% of studies published the entire set of controlled clinical terminology terms required to implement the EHR-derived phenotypes used. Similar patterns were discovered in a review of over 400 biomedical research studies with only a single study making a full protocol available [17]. With the volume and breadth of scientific output using EHR data steadily increasing [19], this nonreproducibility could potentially hinder the pace of translation of research findings.

No common agreed and accepted definition of *reproducibility* currently exists across scientific disciplines. Semantically similarly concepts such as “replicability” and “duplication” are often used interchangeably [20]. In this work, we define *reproducibility* as the the provision of sufficient methodological detail about a study so it could, in theory or in actuality, be exactly repeated by investigators. In the context of EHR research, this would involve the provision of a detailed (and ideally machine-readable) study protocol, information on the phenotyping algorithms used to defined study exposures, outcomes, covariates and populations and a detailed description of the analytical and statistical methods used along with details on the software and the programming code. This in turn, will enable independent investigators to apply the same methods on a similar dataset and attempt to obtain consistent results (a process often referred to as *results replicability*).

### Electronic health record analytical challenges

**Fig. 1:**
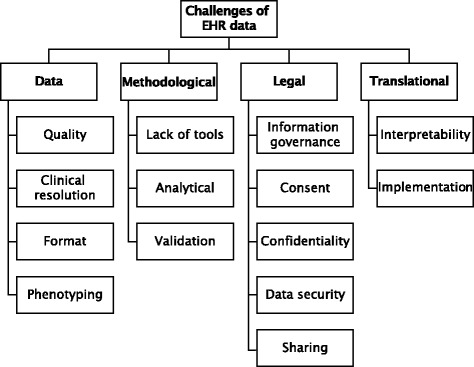
Analytical challenges associated with using big health data for biomedical research span the methodological, ethical, analytical and translational domains

EHR data can broadly be classified as: a) structured (e.g. recorded using controlled clinical terminologies such as as the International Classification of Diseases—10th revision (ICD-10) or Systematic Nomenclature of Medicine – Clinical Terms (SNOMED-CT [21]), b) semi-structured (e.g. laboratory results and prescription information that follow a loose pattern that varies across data sources), c) unstructured (e.g. clinical text [22]) and d) binary (e.g. medical imaging files). Despite the numerous advantages EHR data offer, researchers face significant challenges (Fig. 1).

A primary use-case of EHR data is to accurately extract phenotypic information (i.e. disease onset and progression), a process known as *phenotyping*, for use in observational and interventional research [5]. Phenotyping however is a challenging and time-consuming process as raw EHR data require a significant amount of preprocessing before they can be transformed into research-ready data for statistical analyses [23]. The context and purpose in which data get captured (e.g. clinical care, audit, billing, insurance claims), diagnostic granularity (e.g. post-coordination in SNOMED-CT vs. fixed-depth in ICD) and data quality vary across sources [24].

**Fig. 2:**
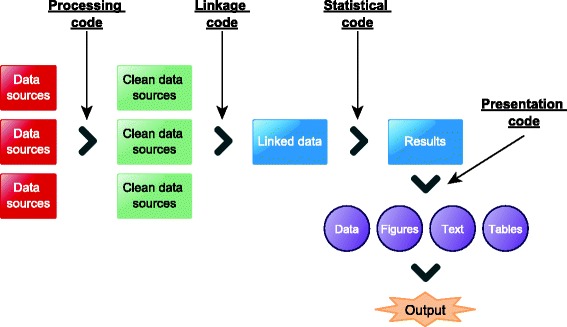
A generic EHR analytical pipeline can generally be split into several smaller distinct stages which are often executed in an iterative fashion: 1) raw EHR data are pre-processed, linked and transformed into statistically-analyzable datasets 2) data undergo statistical analyses and 3) scientific findings are presented and disseminated in terms of data, figures, narrative and tables in scientific output

EHR data preprocessing however, is not performed in a reproducible and systematic manner and as a result, findings from research studies using EHR data potentially suffer from poor reproducibility. Phenotyping algorithms defining study exposures, covariates and clinical outcomes are not routinely provided in research publications or are provided as a monolithic list of diagnostic terms but often miss critical implementation information. For example, a phenotyping algorithm using diagnostic terms in hospital care should consider whether a term is marked as the primary cause of admission or not but this important distinction is often ommited from manuscripts. Common data manipulations (Fig. 2) on EHR datasets are repeated *ad nauseam* by researchers but neither programmatic code nor data are systematically shared. Due to the lack of established processes for sharing and cross-validating algorithms, their robustness, generalizability and accuracy requires a significant amount of effort to assess [25]. In genomics for example, cross-referencing annotations of data produced by related technologies is deemed essential [26] (e.g. reference Single Nucleotide Polymorphism (SNP) id numbers, genome annotations), but such approaches are not widely adopted or used in biomedical research using EHR data.

### Electronic health records research reproducibility

Significant progress has been achieved through the establishment of initiatives such as REporting of studies Conducted using Observational Routinely-collected Data (RECORD) [27, 28] and the STrengthening the Reporting of OBservational studies in Epidemiology (STROBE) [29, 30]. RECORD and STROBE are international guidelines for studies conducted using routinely-collected health data. The guidelines focus on the systematic reporting of implementation details along the EHR analytical pipeline: from study population definitions and data linkage to algorithm details for study exposures, covariates and clinical outcomes (Table 1). Often however, researchers lack the tools and methods to actuate the principles behind these guidelines and fail to integrate them into their analytical process from the start but rather try to incorporate them before publication in an ad hoc fashion. This lack of familiarity with best practices around scientific software development tools and methods prevents researchers from creating, maintaining and sharing high-quality EHR analytical pipelines enabling other researchers to reproduce their research.

**Table 1:** REporting of studies Conducted using Observational Routinely collected Data (RECORD) recommendations on reporting details around EHR algorithms used to define the study populations, exposures and outcomes

RECORD guideline principle	Description
id number	
6.1	The methods of study population selection (such as codes or algorithms used to identify subjects) should be listed in detail.
7.1	A complete list of codes and algorithms used to classify exposures, outcomes, confounders, and effect modifiers should be provided.
13.1	Describe in detail the selection of the persons included in the study (i.e., study population selection) including filtering based on data quality, data availability and linkage.
22.1	Authors should provide information on how to access any supplemental information such as the study protocol, raw data or programming code.

We argue that EHR research can greatly benefit from adopting practices used in adjunct scientific disciplines such as computer science or computational biology in order to reduce the potential irreproducability of research findings using such complex data sources. In this manuscript, we review and identify a series of methods, tools and approaches used in adjacent quantitative disciplines and make a series of recommendations on how they can be applied in the context of biomedical research studies using EHR. These can be used to potentially address the problem of irreproducibility by enabling researchers to capture, document and publish their analytical pipelines. Where applicable, we give examples of the described methods and approaches in R. Adopting best-practices from scientific software development can enable researchers to produce code that is well-documented, robustly tested and uses standardized programming conventions which in turn extend its maintainability. The primary audience of our work is the increasingly expanding cohort of *health data scientists*: researchers from a diverse set of scientific backgrounds (such as for example clinicians or statisticians) that have not been exposed to formal training in computer science or scientific software development but are increasingly required to create and use sophisticated tools to analyze the large and complex EHR datasets made available for research.

## Methods and results

We searched published literature, gray literature and Internet resources for established approaches and methods used in computer science, biomedical informatics, bioinformatics, computational biology, biostatistics, and scientific software engineering. We evaluated and described the manner in which they can be used for facilitating reproducible research using EHR and address the core challenges associated with this process. Reproducibility has been identified as a key challenge and a core value of multiple adjunct scientific disciplines e.g. computer science [31–33] bioinformatics [34], microbiome research [35], biostatistics [36], neuroimaging [37] and computational biology [38].

We identified and evaluated the following methods and approaches (Table 2):

**Table 2:** Methods and approaches that can enable the reproducibility of biomedical research findings using electronic health records

Method/approach	Recommendations
Scientific software engineering principles	Create generic functions for common EHR data cleaning and preprocessing operations which can be shared with the community
	Produce functions for defining study exposures, covariates and clinical outcomes across datasets which can be maintained across research groups and reused across many research studies
	Create modules for logically grouping common EHR operations e.g. study population definitions or datasource manipulation to enable code maintainability
	Create tests for individual functions and modules to ensure the robustness and correctness of results
	Track changes in analytical code and phenotypt definitions using controlled clinical terminology terms by making use of a source code revision control system
	Use formal software engineering best-practices to document workflows and data manipulation operations
Standardized analytical approaches	Build and distribute libraries for common EHR data manipulation or statistical analysis and include sufficient detail (e.g. command line arguments) for all tools used
	Produce and annotate machine-readable EHR phenotyping algorithms that can be systematically curated and reused by the community
	Use Digital Object Identifiers (DOIs) for transforming research artifacts into shareable citable resources and cross-reference from research output
	Deposit research resources (e.g. algorithms, code) in open-access repositories or software scientific journals and cross-reference from research output
	Virtual machines can potentially be used to encapsulate the data, operating system, analytical software and algorithms used to generate a manuscript and where applicable can be made available for others to reproduce the analytical pipeline.
Literate programming	Encapsulate both logic and programming code using literate programming approaches and tools which ensure logic and underlying processing code coexist

1. *Scientific software engineering principles*: modular and object oriented programming, test-driven development, unit testing, source code revision control;
2. *Scalable analytical approaches*: standardized analytical methods, standardized phenotyping algorithms and
3. *Literate programming*

### Scientific software engineering

The nature and complexity of EHR data often requires a unique and diverse set of skills spanning medical statistics, computer science and informatics, data visualization, and clinical sciences. Given this diversity, it’s fair to assume that not all researchers processing and analysing EHR data have received formal training in scientific software development. For the majority of researchers, unconscious practices can creep into the developed code, which if never made publicly available, will never be discovered and yet underpin most published scientific claims. No researcher wants to be put into the position of retracting their manuscript from a journal or having to contact a scientific consortium to ask they repeat months of analyses due to an error discovered in their analytical code. While these issues are not unique in EHR research, they are amplified given its multidisciplinary nature.

There is a subtle but prevalent misconception that analytical code does not constitute *software* as it’s written for a statistical package (e.g. R [39] or Stata [40]) and not in a formal programming language (e.g. Python [41] or Java [42]). As a result, the majority of researchers inadvertently fail to acknowledge or adopt best-practice principles around scientific software engineering. This could not be further from the truth as, by definition, code written for transforming raw EHR into research-ready datasets and undertaking statistical analyses is both complex and sophisticated due to the inherent complexity and heterogeneity of the data. While not directly a technical solution, facilitating scientists to obtain up to date training in best practices through training initiatives such as Software Carpentry [43], can potentially enable them to produce better quality code.

There is no optimal manner in which scientific software can be developed for tackling a particular research question as this is intrinsically an extremely problem-dependent set of tasks. Adopting scientific software engineering best practices however can provide EHR researchers with the essential bedrock of producing, curating and sharing high-quality analytical code for re-use by the scientific community. In general, scientific code development can be divided into several different phases which are usually executed and evaluated in smaller, rapid iterations: planning, coding, testing, debugging, documentation and analysis. In each of these phases, a number of tools and methods exist that enable researchers to manage provenance, readability and usability of their code. We review several of the most critical ones: modular programming, test-driven development and source code revision control in sections below.

#### Modular and object oriented programming

Adopting a modular programming approach will allow EHR researchers to organize their code more efficiently and subsequently enable its documentation and re-use, both by them and other researchers. Modular programming is essentially a software design technique that emphasizes separating the functionality of a program into smaller, distinct, independent and interchangeable modules [44]. This translates to splitting code (that is often produced as a single, large, monolithic file) into several smaller modules that contain similar operations or concepts and that in turn can be stored as independent source code files. These modules can contain anything from a collection of functions to process a particular set of data fields (e.g. convert laboratory test measurements from one measurement unit to another) or entire modules dealing with the intricacies of one particular data source (e.g. extract and rank the causes of hospitalization from administrative data).

Common EHR data transformation or analysis operations (Table 3) can be created as functions which can then be shared across modules. Defining functions that can be repeated across different modules significantly reduces the complexity and increases the maintainability of code. The majority of software applications used in EHR research allow both the sourcing of external files and libraries. For example, in R, the *source* command sources an external R file and the *library* command loads an external library into the current namespace. Functions can be easily defined using the *function* command.

**Table 3:**
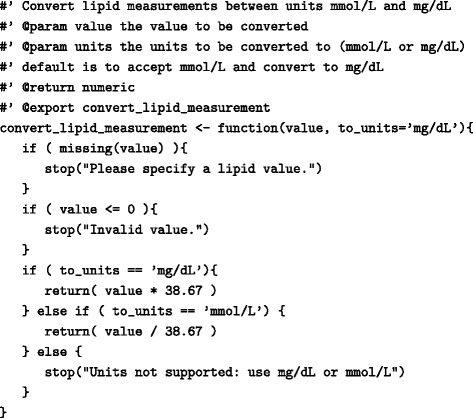
Example of an R function for converting lipid measurements between mmol/L and mg/dL units

Function arguments (value and units) are validated prior to performing the calculation and an error is raised if incorrect or missing parameters are supplied

**Fig. 3:**
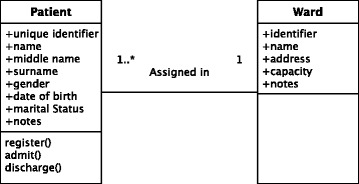
Simple example of a Unified Modelling Language (UML) class diagram Class diagrams are static representations that describe the structure fo a system by showing the system’s classes and the relationships between them. Classes are represented as boxes with three sections: the first one contains the name of the class, the second one contains the attributes of the class and the third one contains the methods. Class diagrams also illustrate the relationships (and their multiplicity) between different classes. In this instance, a patient can be assigned to a single ward within a hospital whereas a ward can have multiple patients admitted at any time (depicted as 1..*)

Adjacent to modular programming is the concept of object oriented programming (OOP) [45]. OOP is a software programming approach based on the concept of *objects* which contains both data (*attributes*) and procedural code (*methods*) to work on the data and can interact with other objects. Formal definitions of objects (i.e. available attributes and methods) are provided by *classes* and *objects* themselves are instances of classes. Central to OOP is the concept of *encapsulation* which abstracts the data and methods of an object from other objects which are only allowed to interact with them through a predefined template called an *interface*. Interfaces in OOP are a paradigm which allows the enforcement of certain predefined properties on a particular class object. Finally, the concept of *inheritance* allows objects to be organized in a hierarchical manner where each level defined a more specific object than the parent level and inherits all the attributes and methods of the parent level. Methods of classes can have a number of preconditions and postconditions defined i.e. predicates that must always hold true just before or right after the execution of a piece of code or the execution is invalidated [46]. Finally, formal software design modelling languages, such as the Unified Modelling Language (UML), [47] can assist researchers in designing and visualizing complex software applications and architectures. Furthermore, modelling languages can be used as a common point of reference and communication across multidisciplinary EHR research groups as they provide non-technical, unambigious graphical representations of complex approaches. An example of a very simple UML class diagram is provided in Fig. 3.

#### Test-driven development and unit testing

Test-driven development (TDD) is a software development approach where automated tests are created prior to developing functional code [48]. In addition to testing, TDD involves writing automated tests of a program’s individual units, a process defined as *unit testing* [49]. A unit is the smallest logical component of a larger software application that can be tested. The majority of tools and languages used in EHR research have some mechanism to directly facilitate code testing. For example, in Stata code testing can be implemented using the *testcase* class in Mata while in SAS [50] unit testing is facilitated through the FUTS [51] or SASUnit [52] libraries. TDD can enable complex EHR pre-processing and analytical code to be adequately and thoroughly tested iteratively over the lifetime of a research project.

Several R libraries exist (e.g. testthat [53], RUnit [54] and svUnit [55]) that enable researchers to create and execute unit tests. RUnit and svUnit are R implementions of the widely used JUnit testing framework [56] and contain a set of functions that check calculations and error situations (Table 4). Such tests can then be integrated within a continuous integration framework [57], a software development technique that enables the automatic execution of all tests whenever an underlying change in the source code is made and in a way ensures that errors are detected earlier. More advanced methods, such as executing formal software verification methods [58] against a predefined specification can be useful for larger and more complex projects.

**Table 4:**
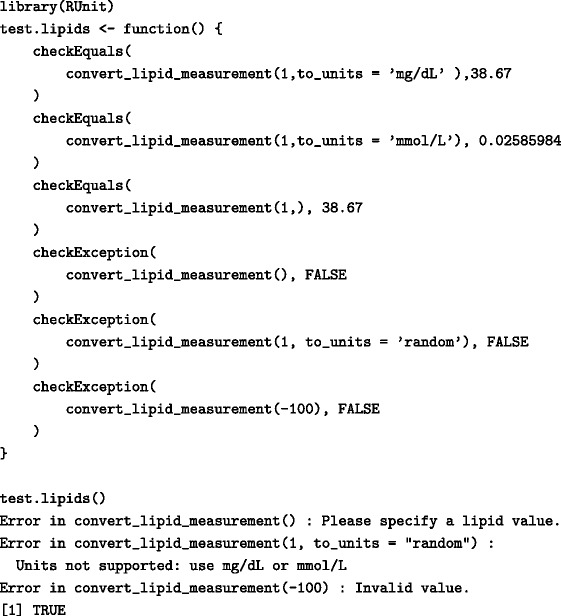
Using the RUnit library to perform unit tests for a function converting measurements of lipids from mmol/L to mg/dL

Combinations of valid, invalid, and missing function parameters are tested and the output returned from the function is examined

#### Source code revision control

**Fig. 4:**
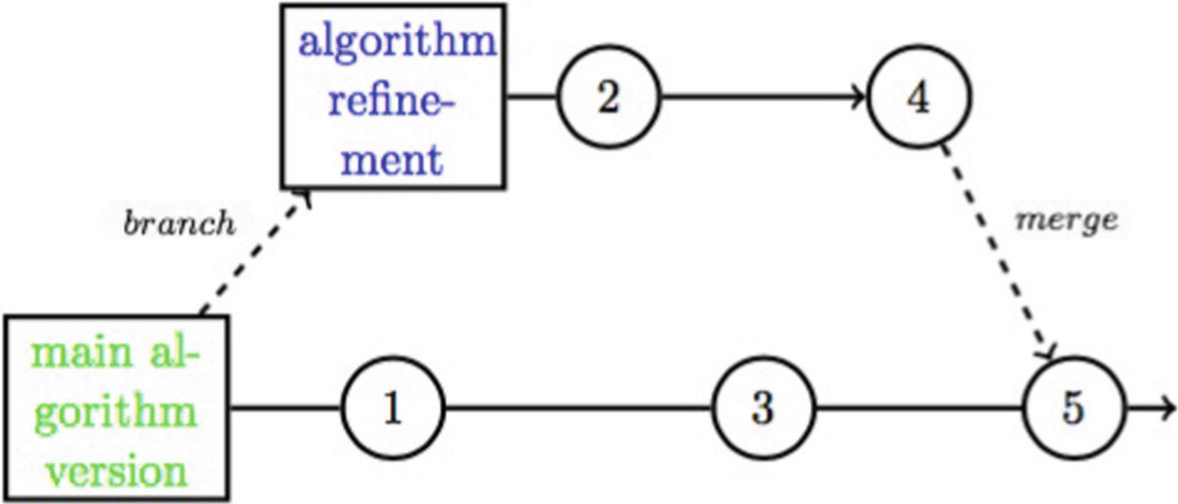
Example of an algorithm managed by version control software Example of an algorithm managed by version control software. The master algorithm version is located on the main development line that is not on a branch, often called a trunk or master, is in green. An individual refinement branch, currently being worked on without affecting the main version is in green and is eventually merged with the main development version [95]

EHR research invariably generates a substantial amount of programming code across the entire EHR pipeline. Even minor changes, accidental or intended (e.g. updating a disease exposure definition), in the code can have large consequences in findings. Given the collaborative and iterative nature of EHR research, it is essential for researchers to have the ability to track changes in disease or study population definitions over time and share the code used in a transparent manner. The standard solution for tracking the evolution of code over time is to use a version control system (Table 5) such as Git [59] or subversion [60]. Version control systems, widely used in software engineering, are applications that enable the structured tracking of changes to individual text-based files both over time and across multiple users [61]. Version control also enables *branching*: the duplication of the core code for the purpose of parallel development independent of the parent code-base. Branching enables the isolation of code for the purposes of altering or adding new functionality or implementing different approaches. In the case of phenotyping algorithms, several branches of the same project may contain alternate implementations of the algorithm with the same core features but slight variations. If only one approach is needed in the end, the relevant branch can then be merged back into the main working code-base (Fig. 4). The use of version control systems in EHR research can enable researchers to keep versioned implementations of exposure, outcomes and phenotype definitions and document the reasons for any changes over time. Computable versions of phenotyping algorithms [62] can also be stored within a version control system for the same reasons.

**Table 5:**
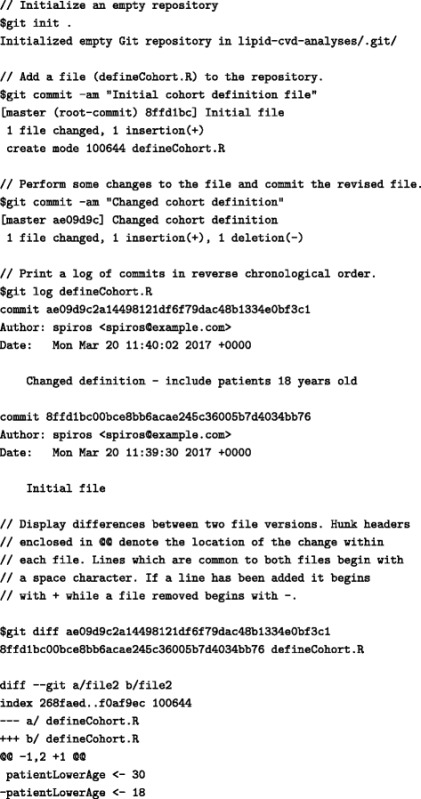
Example of using git to initialize an empty repository and track changes in a versioned file defining a study cohort

Phenotyping algorithms defining disease cases and controls are often developed iteratively and refined when new data become available or changes in the underlying healthcare process model cause the data generation or capture process to change. In the CALIBER EHR research platform for example [3], phenotyping algorithms and their associated metadata are stored and versioned in a private version control system. This includes the actual SQL code for querying the raw data, the implementation details and logic of the algorithm, the diagnostic terms and their relative position used and any other relevant metadata (such as author, date of creation, date of validation) in a bespoke text-based format. This enables researchers to keep track of changes of definitions at the desired time granularity and facilitates the collaborative creation of algorithms. The metadata and implementation details are then made available through the CALIBER Data Portal [63] for other researchers to download and use.

### Scalable analytical approaches

#### Standardized analytical methods

Scientific software is often at first developed behind closed doors and public release is only considered around or after the time of publication [64]. The standardization of common analytical approaches and data transformation operations in EHR research will potentially enable the reproducibility of scientific findings and fuel a sustainable community around the use of EHR data for research. Adjunct scientific disciplines have adopted this principle through the creation of large software libraries that contain a variety of common analytical approaches [65, 66]. For example, Bioconductor [67] was established in 2001 as an open source software for performing bioinformatics operations based on R. It serves as a common software platform that enables the development and implementation of open and accessible tools. Bioconductor promotes high quality documentation, and enables standard computing and statistical practices to produce reproducible research. The documentation across sections of each project is clear, accurate and appropriate for users with varying backgrounds on the programming languages and analytic methods used. There is particular emphasis on programming conventions, guidance, and version control, all of which greatly benefit from being perfomed in a standardized manner.

Adopting similar approaches in EHR research can arguably allow researchers to use and re-use standardized data cleaning, manipulation and analysis approaches. The reproducibility pipeline may require a more explicit structure where specific analytic workflows are tied to complete processes illustrating the decision tree from data preparation to data analysis. The ability to reproduce biomedical research findings depends on the interconnection of stages such as detailing the data generation processes, phenotyping definitions and justifications, different levels of data access where applicable, specifics on study design (e.g. matching procedures or sensitivity analyses) and the statistical methods used. Building generic and re-usable software libraries for EHR data is challenging due to the complexity and heterogeneity across data sources. While some libraries for manipulating and analysing EHR data exist [68], these are narrowly focused on specific data sources are challenging to generalize across other sources, countries or healhcare systems. Building and curating software libraries following the best practices outlined in this manuscript and disseminating them with standard scientific output is recommended in order to grow and sustain a community of tools and methods that researchers can use. Examples such as Bioconductor can offer inspiration on how to build an active community around these libraries that will facilitate and accelerate their development, adoption and re-use by the community.

A key aspect of developing software tools for data processing is estimating the expected data growth and designing modules and tools accordingly to accommodate future increases in data. Given the steady increase in size and complexity of EHR data, workflow management systems used in bioinformatics such as Galaxy [69], Taverna [70], and Snakemake [71] can enable the development of scalable approaches and tools in EHR research. Workflow management systems enable researchers to break down larger monolithic tasks or experiments into a series of small, repeatable, well defined tasks, each with rigidly defined inputs, run-time parameters, and outputs. This allows researchers to identify which parts of the workflow are a bottleneck or in some cases which parts could benefit from pararellization to increase throughput. They also allow the integration of workload managers and complex queuing mechanisms that can also potentially lead to better management of resources and processing throughput [72]. Pipelines can be built to obtain snapshots of the data, validate using predefined set of rules or for consistency (e.g. against controlled clinical terminologies) and then transform into research-ready datasets for statistical analysis. Such pipelines can then potentially be shared and distributed using container technologies such as Docker [73] or package managers like Conda [74]. Docker is an open-source platform that uses Linux Containers (LXC) to completely encapsulate and make software applications portable. Docker containers require substantially less computational resources than virtual machine-based solutions and allow users to execute applications in a fully virtualized environment using any Linux compatible language (e.g. R, Python, Matlab [75], Octave [76]). Docker libraries can be exported, versioned and archived, thus ensuring that the programmatic environment will be identical across compatible platforms.

Limited backwards compatibility can often hinder the reproducibility of previous scientific findings. For example, newer versions of a statistical analysis tool might not directly support older versions of their proprietary data storage format or an analytical tool might be compiled using a library that is no longer available in newer versions of an operating system. These issues can potentially be mitigated through the use of virtualization [77]. Virtual machines (e.g. Oracle VirtualBox [78], VMware [79]) are essentially containers that can encapsulate a snapshot of the OS, data and analytical pipelines in a single binary file. This can be done irrespective of the “host” operating system that is being used and these binary files are compatible across other operating systems. Other researchers can then use these binary files to replicate the analytical pipeline used for the reported analysis.

#### Standardized phenotyping algorithms

No widely accepted approach currently exists for storing the implementation and logic behind EHR phenotyping algorithms in a machine-readable and transportable format. The translation from algorithm logic to programming code is performed manually, and as a result, is prone to errors due to the complexity of the data and potential ambiguity of algorithms [25]. In their work, Mo and colleagues describe the ideal characteristics of such a format such as the ability to support logical and temporal rules, relational algebra operations, integrate with external standardized terminologies and provide a mechanism for backwards-compatibility [80]. The creation and adoption of computational representations of phenotyping algorithms will enable researchers to define and share EHR algorithms defining exposures, covariates and clinical outcomes and share them in a standardized manner. Furthermore, machine-readable representations of EHR phenotyping algorithms will enable their integration with analytical pipelines and will benefit from many of the approaches outlined in this manuscript such as version control, workflow systems and standardized analytical libraries [62, 81]. Finally, algorithm implementations can also be uploaded in open-access repositories [82] or software journals [83] where they could be assigned a unique Digital Object Identifier (DOI) and become citeable and cross-referenced in scientific output.

### Literate programming

Publishing study data online or in secure repositories alongside the code to preprocess and analyse it may be possible in some biomedical research domains, but is typically not an option for EHR research given the strict information governance restrictions and legal frameworks researchers operate under. Additionally, EHR sources typically contain the entire patient history and all their interactions with health care settings but only a subset of the original data is used and it is therefore equally important to document and disseminate the process of data extraction as well as the post-processing and analysis.

Extracting the appropriate dataset for research involves specifying lists of relevant controlled clinical terminology terms, timing windows for study population and patient phenotypes, and eligibility criteria. The work of defining the extraction criteria is usually performed by data managers in conjunction with domain experts such as clinicians. The algorithm parameters and implementation details are subsequently converted to machine instructions (e.g. SQL), executed and resulting data are usually exported from a relational database. Although the rationale behind the extraction process and the machine readable code should bear equivalent information content, it is extremely difficult for a human reader to understand the underlying logic and assumptions by reading the code itself [80]. It is also very challenging to fully reproduce the extraction using only the human readable instructions of the agreed protocol given the ambiguity of algorithms and the complexity of the data. Similar challenges exist for the preprocessing of EHR data, such as the definition of new covariates and clinical outcomes, as well as for the analysis and post-processing (such as plotting) of the extracted dataset and results.

**Fig. 5:**
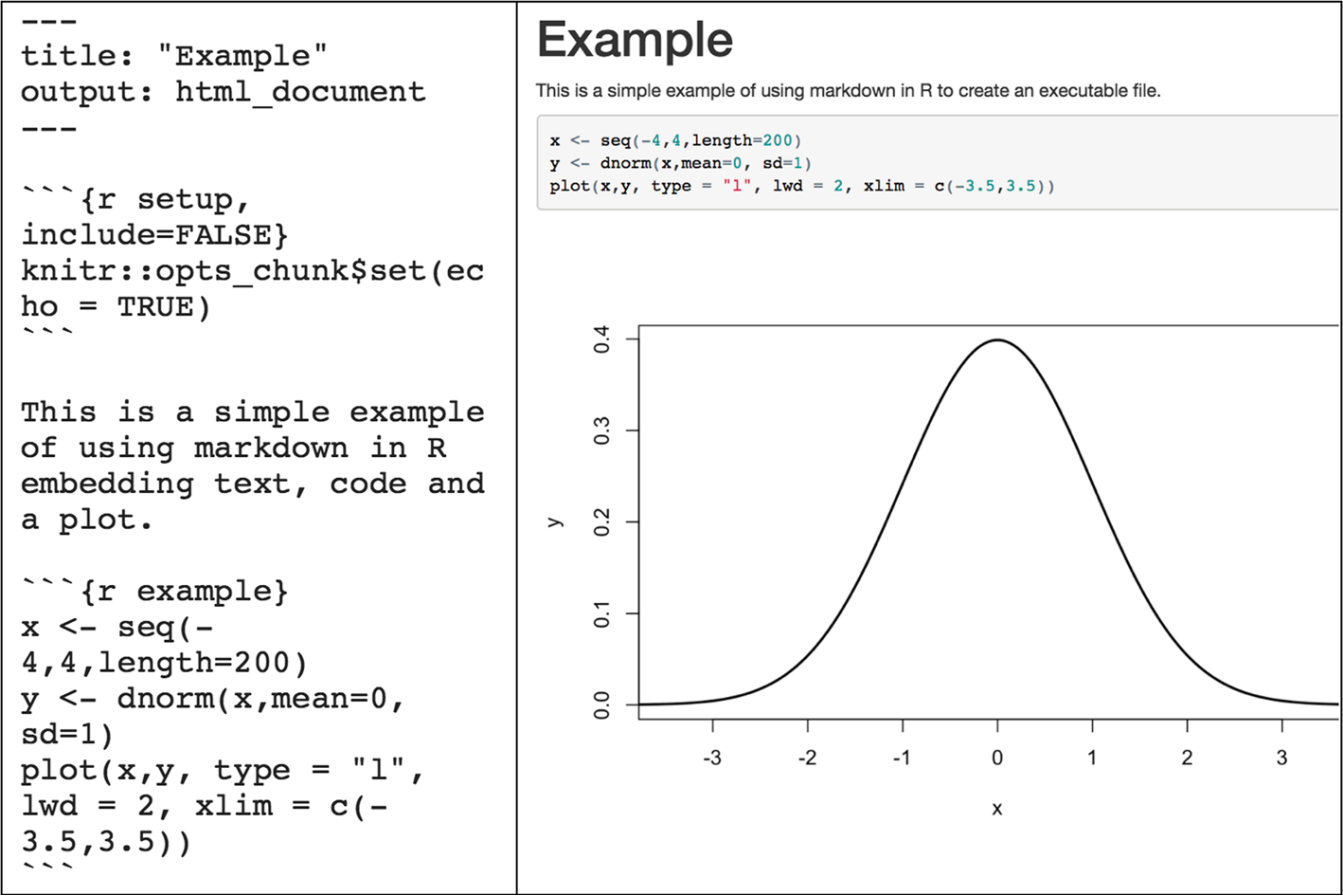
Example of using the Knitr R package to produce a dynamic report with embedded R code and results including a plot. Documentation and data processing code chunks are written in plain text in a file that is processed as RMarkdown. At the top of the file, a series of key: value pair statements in YAML set document metadata such as the title and the output format. Code chunks are enclosed between ``` characters and executed when the document is compiled. Parameters such as *echo* and *display* can be set to specify whether the results of executing the code or whether the actual code itself is displayed. Example taken from http://jupyter.org/

A simple and time-honoured solution to this challenge is the provision of documentation alongside the code used for the extraction/preprocessing/analysis of EHR data. This approach however is often problematic as documentation can often be out-of-date with regards to the code, might be incomplete as it is often written after the analysis is finished and for large projects, linking the correct pieces of documentation to the specific locations in the code can be cumbersome. A potential approach to solving this challenge is *literate programming*. The concept of literate programming was introduced by Donald E Knuth [84] and is not limited to a specific analytical tool or programming language. Literate programming is the technique of writing out program logic in a human language with included (separated by a primitive markup) code snippets and macros. In practice, both the rational behind the data processing pipeline as well as the processing code itself are authored by the user using an appropriate integrated development environment (IDE). The resulting plain text document subsequently undergoes two processes, the first in which the code itself is executed (often referred to as *tangling*) and one in which the formatted documentation is produced (often referred to as *weaving*). The result is a well formatted rich text document, for example Hypertext Markup Language (HTML), which can often include the output of the executed code (e.g. plots, summary tables, analysis results) alongside the original code snippets and documentation (Fig. 5). 

The most popular modern day literate programming tool in R is roxygen [85] while popular report generation packages are Knitr [86] and Sweave [87]. Another widely used tool is Jupyter [88], which is often used with (but not limited to) Python. For example, Johnson et al. published all code necessary to reproduce the data description of the MIMIC-III [89] database in the form of Jupyter notebooks on a GitHub repository [90]. The use of Jupyter notebooks was encouraged, and a specially developed software platform that integrated with notebooks was provided for a datathon using the MIMIC-III database, rendering all resulting analysis fully reproducible [91]. Most EHR research analytical tools support literate programming (Table 6) and its use can greatly facilitate closing the gap between code and narrative.

**Table 6:** Examples of packages and libraries supporting literate programming and report generation in popular analytical/statistical software packages

Statistical/Analytical tool	Relevant packages
R	RMarkdown, Knitr, Sweave, Roxygen
Stata	MarkDoc, Weaver, Ketchup
Python	Jupyter Notebook, Doxygen
Matlab	Doxygen (limited)
Octave	Doxygen [96]
SAS	SASWeave [97], StatRep

Taking the literate programming paradigm ever further, *compendia* [92] are containers for distributing and disseminating the different elements that comprise a piece of computational research. These elements are also fundamental in the concept of literate programming, however in the case of compendia, the data are also contained in the output [93].

## Conclusion

The challenge of reproducibility in science has been widely recognized and discussed [94]. Scientists using EHR data for biomedical research face a number of significant challenges which are further amplified due to the complexity and heterogeneity of the data sources used and the cross-disciplinarity of the field. It is crucial for researchers to adopt best-practices across disciplines in order to enable the reproducibility of research findings using such data. In this manuscript we identify and present a set of principles, methods and tools from adjunct scientific disciplines that can be utilized to enable reproducible and transparent biomedical research using EHR. Enabling reproducible research using EHR is an ongoing process that will greatly benefit the scientific and wider community.
